# Silybin Cocrystals with Improved Solubility and Bioavailability

**DOI:** 10.3390/ph18010090

**Published:** 2025-01-13

**Authors:** Bingqing Zhu, Zhenfeng Ding, Xiaoyi Rong, Shengqiang Li, Xuefeng Mei

**Affiliations:** Pharmaceutical Analytical & Solid-State Chemistry Research Center, Shanghai Institute of Materia Medica, Chinese Academy of Sciences, 555 Zuchongzhi Road, Shanghai 201203, China; zhubingq@simm.ac.cn (B.Z.); rongxiaoyi@simm.ac.cn (X.R.);

**Keywords:** silybin, cocrystal, solubility, bioavailability, L-proline

## Abstract

**Backgroud/Objectives:** Silymarin, an extract from milk thistle, is widely recognized for its therapeutic potential in treating liver disorders. However, its clinical utility is limited by the poor solubility and low bioavailability of its key active ingredient, Silybin. In this study, we sought to address this issue through the development of a novel cocrystal of Silyin. **Methods:** Silybin-L-proline cocrystal was synthesized and the physicochemical properties of the cocrystal were characterized by PXRD, TGA, DSC, and FTIR. Dissolution tests were conducted in various pH solutions, and the impact of precipitation inhibitors was evaluated. Furthermore, pharmacokinetic study in rats were performed to assess the bioavailability. **Results:** The dissolution studies demonstrated that the cocrystal has a significant improvement in dissolution performance, particularly in acidic environments. Furthermore, the use of precipitation inhibitors, such as PVP, prolonged the supersaturation period for adequate absorption. Pharmacokinetic studies in rats revealed that the cocrystal exhibited a 16-fold increase in bioavailability compared to the raw Silybin extract, outperforming the commercial Silybin–phosphatidylcholine complex. **Conclusions:** The Silybin–L-proline cocrystal significantly enhances dissolution and bioavailability, indicating its potential to improve the therapeutic efficacy of Silybin in clinical applications.

## 1. Introduction

Silymarin, extracted from the seeds of milk thistle (*Silybum marianum*), is a complex mixture of flavonolignans and flavonoid polyphenolic compounds. The main silymarin flavonolignans are Silybin, isosilybin, silydianin, and silychristin, among which Silybin stands out as a key active ingredient [1]. Silymarin is well-recognized for its therapeutic liver-protecting activity, which is attributed to its antioxidant, antifibrosis, detoxification, and hepatocyte-protection properties [2,3]. There have been many clinical studies that report the benefits of silymarin in treating various liver disorders [4], such as viral hepatitis [5,6], alcoholic liver disease [7], and nonalcoholic liver disease [8,9].

**Scheme 1:**
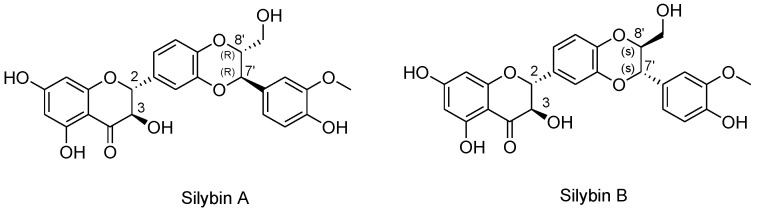
The chemical structure of Silybin A and Silybin B.

Silybin, as the major active constitute of silymarin, exists in two isomeric forms, Silybin A and Silybin B (Scheme 1). They are differentiated with respect to reference positions C-7′ and C-8′. Specifically, Silybin A has a configuration of 2R, 3R, 7′R, 8′R and Silybin B has a configuration of 2R, 3R, 7′S, 8′S. According to one reference [10], although both Silybin A and Silybin B are bioactive, Silybin A has higher bioavailability than Silybin B. Despite the significant therapeutic potential of Silybin, its clinical efficacy is limited by its poor solubility in water (<0.04 mg/mL) and consequently by its low absolute bioavailability (<1%). This challenge has spurred the development of various technological approaches aimed at improving the solubility [11,12] and absorption of Silybin [13], such as self-microemulsifying drug delivery system [14], solid dispersions [15], nanocrystals [16], liposomes [17], and phospholipid complexes [18]. Among these, a phosphatidylcholine complex of Silybin (SILIPHOS^®^) has been commercialized, and it has been utilized in many dietary supplements for liver protection. The complex contains about 30% Silybin. It has been reported that a phosphatidylcholine complex in oily-medium, soft-gel capsules displays 9.6 times higher bioavailability compared with conventional Silybin tablets [18]. Therefore, the recommended dosage of the complex is only 80–160 mg (equivalent to 24–48 mg of Silybin), which is much lower than the typical Silybin dosage of 140 mg. However, the melting point of this complex is only about 35 °C, which may bring up a new challenge for various solid formulations.

Cocrystal technology has emerged as a promising method to enhance the pharmacokinetic profiles of poorly soluble compounds without altering their molecule structures [19,20,21]. Compared to other formulation techniques, cocrystal technology has its unique characteristics and advantages. Specifically, while self-microemulsifying delivery system and liposomes are typically solution-based dosage forms, cocrystals are generally a solid-based dosage form. While nanocrystals have issues with aggregation and solid dispersion can face recrystallization problems, cocrystals are typically more thermodynamically stable. Chen Jianxin et al. have reported three cocrystals of Silybin with salicylic acid, syring acid, and salicylamide, and the cocrystals presented with improved solubility in water. However, the in vivo bioavailability was not investigated [22]. In this work, we expect to explore not only the solubility but also the bioavailability of Silybin cocrystal. A wide range of amino acids, as well as other coformers containing amide or amine groups, were screened. After screening, a novel cocrystal of Silybin with L-proline was obtained. L-proline is a biologically and pharmaceutically significant protogenic non-essential amino acid, which can be regarded as a safe coformer for cocrystallization [23]. In this work, the physicochemical properties were comprehensively evaluated for this cocrystal and a bioavailability study was also conducted. Future work would focus on the investigation of methods for improving its efficacy.

## 2. Results and Discussion

### 2.1. Cocrystal Preparation and PXRD Analysis

**Figure 1:**
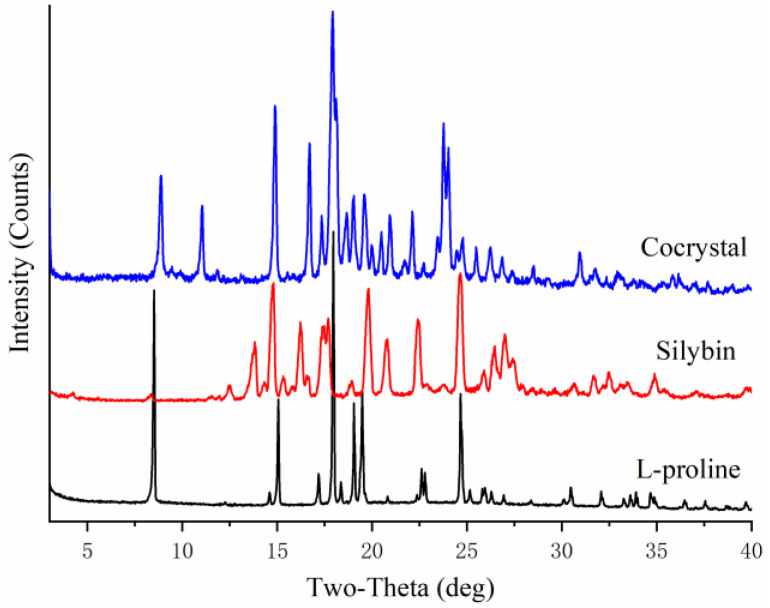
PXRD patterns of Silybin, L-proline, and their cocrystal.

In this work, a novel cocrystal of Silybin with L-proline was obtained. The overlay PXRD pattern of Silybin, L-proline, and their cocrystal is shown in Figure 1 and the peak values are labeled in Figure S1. The cocrystal displays distinct PXRD patterns with Silybin and L-proline. New characteristic diffraction peaks were observed at 2θ = 9.0°, 11.2°, 14.9°, 16.7°, 18.1°, and 19.8°. These peaks indicate the presence of a new phase.

### 2.2. Stoichiometric Ratio Determination

The single crystal structure of this cocrystal was unfortunately not able to be obtained. Therefore, the stoichiometric ratio of Silybin to L-proline in the cocrystal was determined by ^1^H-NMR and HPLC. The ^1^H-NMR spectra of the cocrystal is shown in Figure S2. The peak areas (normalized) at 3.89 ppm corresponding to Silybin and 1.99 ppm corresponding to L-proline indicate that the stoichiometric ratio of Silybin to L-proline is 1:2. This ratio was further validated by an HPLC assay of the cocrystal, which revealed a Silybin content of 67.7%. The consistency between the NMR-derived ratio and the HPLC assay results supports the accuracy of the determined stoichiometric ratio and confirms the composition of the cocrystal. Additionally, the ratio of Silybin A and Silybin B in the cocrystal can also be identified by HPLC. The HPLC pattern of the cocrystal is shown in Figure S3. The signals at 7.030 min and 7.193 min are representative of Silybin A and Silybin B. Using Silybin A and Silybin B as working standards, the ratio of these two isomers in the cocrystal was determined to be 1:1. This indicates that both Silybin A and B can be cocrystallized with L-proline.

### 2.3. FTIR Spectroscopy

**Figure 2:**
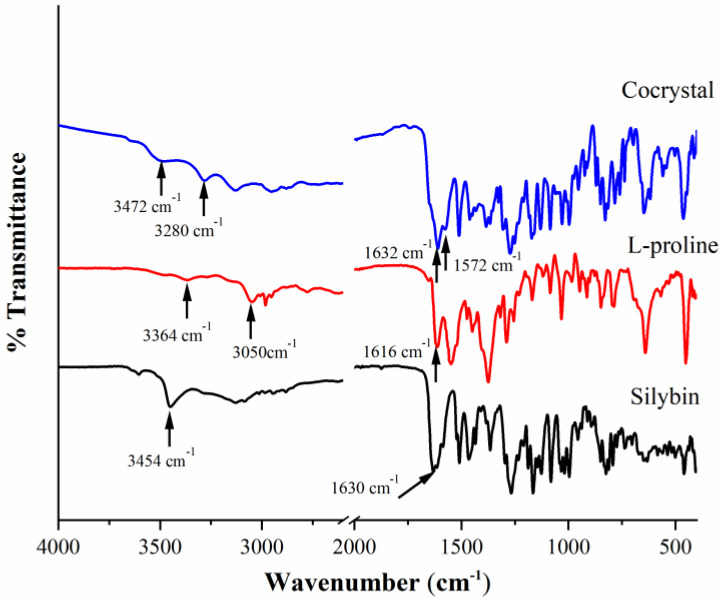
FTIR spectra of Silybin, L-proline, and their cocrystal.

The overlay FTIR spectra of Silybin, L-proline, and their cocrystal are shown in Figure 2. The FTIR spectra of Silybin displays characteristic signals at 3454 cm^−1^ and 1630 cm^−1^, corresponding to the vibration frequencies of the phenolic O–H group and C=O group. For L-proline, the vibration peak of the N–H, O–H, and C=O groups appear at 3364 cm^−1^, 3050 cm^−1^, and 1616 cm^−1^, respectively. In the cocrystal of Silybin and L-proline, the vibration peak corresponding to the O–H group in Silybin was blue-shifted from 3454 cm^−1^ to 3472 cm^−1^. The vibration peak corresponding to the N–H group in L-proline was red-shifted from 3364 cm⁻^1^ to 3280 cm⁻^1^, and the C=O group was red-shifted from 1616 cm⁻^1^ to 1572 cm⁻^1^. However, the vibration of the C=O group in Silybin showed no significant changes. These findings suggest that the –NH and –COOH groups of L-proline, along with the –OH group of Silybin, may be involved in intermolecular hydrogen bond formation in the cocrystal.

### 2.4. Thermal Analysis

**Figure 3:**
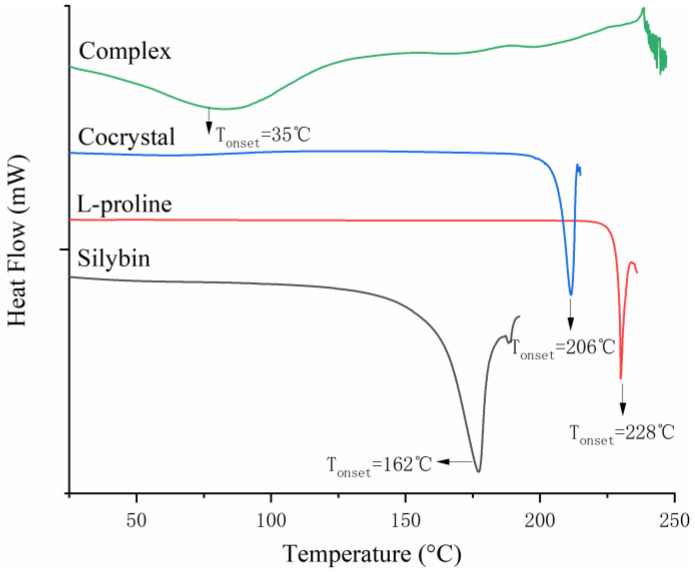
DSC profiles of Silybin, L-proline, Silybin complex, and their cocrystal.

The TGA profile is shown in Figure S4. There was no significant weight loss prior to the decomposition temperature of approximately 200 °C. DSC was employed to further investigate the thermal behavior. As shown in Figure 3, Silybin, L-proline, and their cocrystal exhibit melting peaks at an onset temperature of 162 °C, 109.6 °C, and 206 °C, respectively. However, in the case of the phosphatidylcholine complex, the melting range is quite broad, with the onset of melting occurring around 35 °C and the peak melting point occurring approximately at 84 °C.

### 2.5. Hygroscopicity

**Figure 4:**
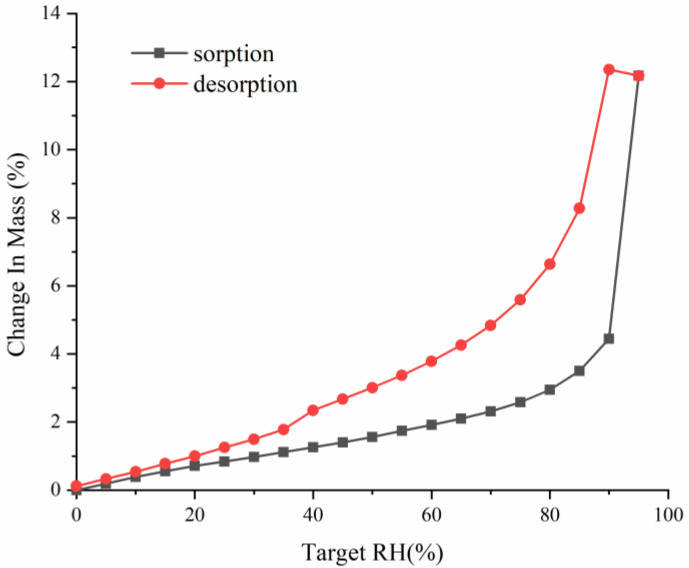
DVS profiles of Silybin cocrystal.

The DVS of the Silybin cocrystal is presented in Figure 4. The water uptake increases continuously with the relative humidity, reaching 2.5% at 40% RH and 3.0% at 80% RH. After the DVS experiment, the cocrystal sample powder was analyzed using PXRD (Figure S5). Although the crystallinity was slightly reduced, the peak position was not changed, which confirmed that it did not undergo dissociation or any phase transformation after a water sorption and desorption cycle.

### 2.6. Powder Dissolution Studies

**Figure 5:**
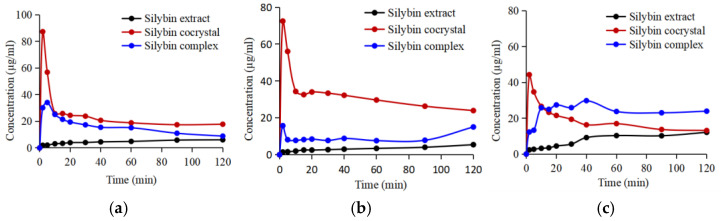
Dissolution profiles of Silybin (black line), Silybin complex (blue line), and Silybin cocrystal (red line) in buffer solutions with (**a**) pH of 2.0, (**b**) pH of 4.5, and (**c**) pH of 6.8.

The dissolution profiles of the raw Silybin, Silybin cocrystal, and Silybin complex were evaluated in buffer solutions with a pH of 2.0, 4.5, and 6.8. The result presents a significant improvement in dissolution performance for both the cocrystal and complex (Figure 5). The peak solubility of the cocrystal consistently occurred at 2 min in all pH conditions, and the apparent solubility of the cocrystal was 44.4, 50.3, and 16.5 times greater than Silybin at a pH of 2.0, 4.5, and 6.8, respectively. This significant improvement can be partly attributed to the high solubility of L-proline in water containing different electrolytes [24,25]. Meanwhile, a “spring-parachute” effect can be observed for the Silybin cocrystal. The supersaturated solution triggers the nucleation and recrystallization of the dissolved Silybin. After 2 h, the solubility of the cocrystal is only 2.9, 4.4, and 1.1 times greater than the raw material. The cocrystal dissociation and the recrystallization of Silybin during the dissolution period was proven by monitoring the PXRD pattern. As is shown is Figure S6, the cocrystal has already converted to raw Silybin after 10 min, which is the reason for the rapid decrease in the dissolution concentration. In the case of the Silybin complex, although its apparent solubility is not as significant as that of the cocrystal, it still presents with 15.3, 10.9, and 5.2 times higher solubility at 2 min and 1.4, 2.8, and 2.0 times higher solubility at 2 h. Finally, their solubilities after being at equilibrium for 24 h are listed in Table S2, which shows that the cocrystal has a similar solubility to the extract.

### 2.7. Powder Dissolution with Precipitation Inhibitors

**Figure 6:**
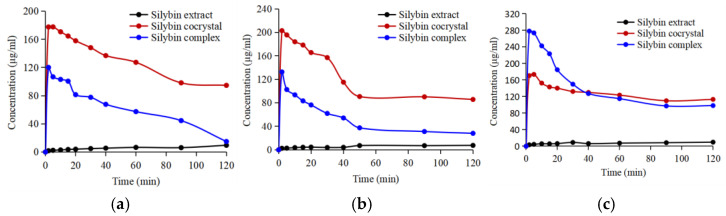
Dissolution profiles of raw Silybin (black line), complex (blue line) and cocrystal (red line) in buffer solutions with (**a**) pH of 2.0 with 0.5% PVP, (**b**) pH of 4.5 buffer with 0.5% PVP, and (**c**) pH of 6.8 buffer with 0.5% PVP.

The previous dissolution study reveals a significant “spring-parachute” effect for the Silybin cocrystal. This behavior indicates that Silybin, in a supersaturated state, readily nucleates and precipitates. Therefore, to maintain the supersaturation advantage of the cocrystal, various polymers, such as pyrroidone (PVP), polyethylene glycol (PEG), and hydroxypropyl methylcellulose (HPMC), were selected as precipitation inhibitors, which could retard the recrystallization of Silybin through numerous mechanisms [26] The dissolution profile at a pH of 2.0 in the presence of polymers is shown in Figure S7. Except for PEG, the polymers are able to inhibit the crystallization of Silybin in solution, wherein PVP demonstrates the most effective inhibition. The supersaturation period of the cocrystal is significantly extended. Furthermore, a dissolution study was conducted in buffers with three different pHs in the presence of PVP. As shown in Figure 6, the apparent solubility of the cocrystal was 77.3, 100.0, and 50.5 times greater than that of raw Silybin at 2 min. Additionally, the supersaturation state can be maintained over a reasonable time period to promote adequate absorption. After 2 h, the solubility is still 11.3, 9.9, and 11.6 times greater than that of raw Silybin. In the case of the Silybin complex, although it shows a higher apparent solubility at a pH of 6.8, its dissolution performance is inferior to the cocrystal in the other two pH solutions. Specifically, it presents with 50.5, 67.7, and 85.4 times higher solubility at 2 min and 3.7, 1.6, and 10.0 times higher solubility at 2 h.

### 2.8. Pharmacokinetic Study

**Figure 7:**
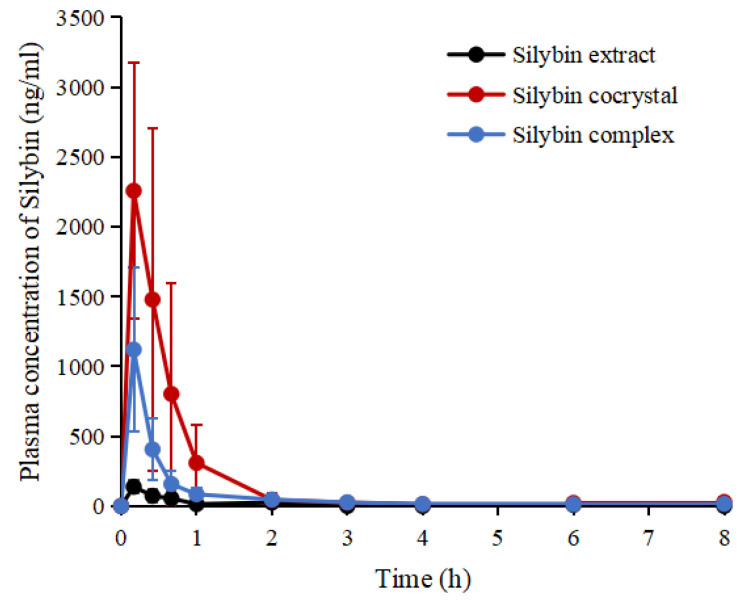
Plasma concentration–time curves of raw Silybin, phosphatidylcholine complex of Silybin, and Silybin cocrystal (data are expressed as mean ± SD, n = 6).

The in vitro dissolution study demonstrates that the cocrystal exhibits significant advantages over the raw material Silybin. To further assess whether the cocrystal can indeed improve Silybin’s bioavailability, a pharmacokinetic study in rats was conducted. In this study, the cocrystal was compared with both raw Silybin and the phosphatidylcholine complex (SILIPHOS^®^). Since the Silybin cocrystal tends to dissociate in a water-based medium, we used soybean oil as the delivery medium. The cocrystal remains unchanged after being suspended in soybean oil for 24 h. The PK curve is shown in Figure 7, and the pharmacokinetic parameters are summarized in Table 1. At the same dosage of 50 mg/kg, both the phosphatidylcholine complex and cocrystal exhibit much higher bioavailability than raw Silybin. The C_max_ and AUC_0–8h_ of the phosphatidylcholine complex are 8.1 and 6.4 times higher than Silybin. The cocrystal improves bioavailability to a greater extent. The C_max_ and AUC_0–8h_ of the cocrystal are 16.4 and 16.2 times higher than Silybin.

## 3. Materials and Methods

### 3.1. Materials

Silybin with a purity greater than 95% was purchased from Liaoning Fengrui Natural Biotechnology Co., Ltd. (Panjin, China). Silybin–phosphatidylcholine complex (SILIPHOS^®^), which contains about 30% Silybin was purchased from Indena Pharmaceuticals (Milano, Italy). L-proline with a purity greater than 98% was purchased from J&K Scientific Ltd. (Beijing, China). All solvents used in this work were purchased from Sinopharm Chemical Reagent Co., Ltd. (Shanghai, China). The specifications of all the chemical samples are listed in Table S1.

### 3.2. Synthesis of Silybin Cocrystal with L-Proline

A total of 100 g of Silybin (0.2 mol) and 47.7 g of L-proline (0.4 mol) were added to 0.9 L of ethanol and stirred at 60 °C for 12 h. The solution was then filtered to obtain cocrystal powder.

### 3.3. Powder X-Ray Diffraction (PXRD)

PXRD patterns were collected using a Bruker D8 Advance X-ray diffractometer (Cu Kα radiation) from Bruker Corporation (Berlin, Germany). The tube voltage and current of the generator were set to 40 kV and 40 mA, respectively. The samples were measured with a continuous scan at 0.1 s step^−1^ in the 3–40° 2θ range with a step size of 0.02°. Data were imaged and integrated with RINT Rapid, and peaks were analyzed with Jade 6.0 from Ragaku (Win-OS V6.2.9200(2)).

### 3.4. Nuclear Magnetic Resonance (NMR) Spectroscopy

NMR experiments were conducted on a Bruker Avance III 600 Spectrometer from Bruker Corporation (Berlin, Germany), operating at a frequency of 600 MHz. The measurements were performed at room temperature. The samples were dissolved in deuterated methanol (MeOH) as the solvent with a weight of approximately 5 mg. The NMR spectra of cocrystal displays signals at 7.13 (dd, *J* = 4.6, 2.0 Hz, 1H), 7.07 (dt, *J* = 8.4, 2.5 Hz, 1H), 7.05–7.01 (m, 2H), 6.93 (dd, *J* = 8.1, 1.9 Hz, 1H), 6.86 (d, *J* = 8.1 Hz, 1H), 5.95 (d, *J* = 2.2 Hz, 1H), 5.92 (t, *J* = 2.2 Hz, 1H), 5.01 (d, *J* = 11.5 Hz, 1H), 4.95 (dd, *J* = 8.1, 1.6 Hz, 1H), 4.55 (dd, *J* = 11.5, 4.3 Hz, 1H), 4.15–4.05 (m, 1H), 3.89 (d, *J* = 1.2 Hz, 3H), 3.73 (dd, *J* = 12.3, 2.5 Hz, 1H), 3.52 (dd, *J* = 12.3, 4.5 Hz, 1H), 3.40 (dt, *J* = 11.5, 6.9 Hz, 2H), 3.25 (dt, *J* = 11.4, 7.3 Hz, 2H), 2.38–2.26 (m, 2H), 2.13 (dq, *J* = 13.2, 6.6 Hz, 2H), and 1.99 (pd, *J* = 7.1, 2.9 Hz, 4H).

### 3.5. Thermogravimetric Analysis (TGA)

TGA experiments were conducted on TGA-55 equipment from TA Instrument (New Castle, DE, USA). Each sample (5–10 mg) was placed in an open aluminum oxide pan and heated from 25 °C to 410 °C at 10 °C min^−1^. Nitrogen was used as the purge gas at a flow rate of 40 mL min^−1^.

### 3.6. Differential Scanning Calorimetry (DSC)

DSC experiments were performed on a PerkinElmer DSC 8500 instrument from PerkinElmer (Waltham, MA, USA). Accurately weighed samples (3–5 mg) were heated in sealed non-hermetic aluminum pans at a heating rate of 10 °C min^−1^ and purged under nitrogen gas flow at a rate of 50 mL min^−1^. Two-point calibration using indium and tin was carried out to check the temperature axis and heat flow of the equipment.

### 3.7. Dynamic Vapor Sorption (DVS)

The hygroscopicity behaviors of the materials were studied on an Intrinsic DVS instrument from Surface Measurement Systems, Ltd. (Wembley, UK). The samples were mounted on a balance and studied over a humidity range from 0 to 95% RH at 25 °C. Each humidity step was conducted if less than a 0.02% weight change occurred within 10 min.

### 3.8. FTIR Spectroscopy

A Nicolet FTIR 750 spectrometer from Thermo Fisher Scientific (Waltham, MA, USA) was used to conduct the FTIR experiments. The FTIR data were collected by a diamond module in a range of 4000 to 400 cm^−1^. The average scan number was 32 and the spectral resolution was 4 cm^−1^.

### 3.9. Powder Dissolution

Non-sink powder dissolution experiments were performed on Silybin and its cocrystal. Excess amounts of 60 mg of Silybin (or corresponding amounts of its cocrystal) were added to a dissolution vessel containing 15 mL of dissolution medium, which are pH of 2.0, 4.5, and 6.8 buffers in the absence or presence of 0.5% polyvinylpyrrolidone (PVP). The dissolution experiments were conducted at 37 °C with a stirring rate of 50 rpm. Sampling was performed at the following time points of 2, 5, 10, 15, 20, 30, 40, 60, 90, and 120 min. At each time point, 0.6 mL of the sample was withdrawn and filtered through a 0.45 µm aqueous filter membrane. The filtrate (300 µL) was then diluted with an equal volume of methanol and analyzed using HPLC.

### 3.10. High-Performance Liquid Chromatography (HPLC)

Silybin was quantified using an Agilent 1260 series from Agilent Technologies, Inc. (Santa Clara, CA, USA), equipped with a quaternary pump (G1311C), a diode-array detector (G1315D) set to 288 nm, and a 4.6 × 150 mm, 5 μm Agilent Eclipse Plus C18 column. The mobile phase consists of water (0.5% acetic acid) and methanol (48:52, *v*/*v*) and was run at a flow rate of 1.0 mL min^−1^. The column temperature was set to 25 °C, and the injection volume was 10 μL.

### 3.11. Pharmacokinetic Study

Pharmacokinetic experiments were conducted in male Sprague Dawley rats to compare the bioavailability of raw Silybin extract, Silybin cocrystal, and Silybin–phosphatidylcholine complex. The experimental protocol was approved by the Institutional Animal Care and Use Committee of Shanghai Institute of Materia Medica and conformed to the Guide for Care and Use of Laboratory Animals. The IRB number is 2023-08-MXF-07. Silybin extract, Silybin cocrystal, and Silybin–phosphatidylcholine complex were previously sieved through 100-mesh sieves. The resulting powders were then dispersed in soybean oil. Eighteen male rats weighing 220–250 g were randomly allocated into three groups (six rats in each group) with free access to water and overnight fasting before administration. Each group was administered a dose of 50 mg/kg (equivalent to Silybin) by gavage, and feed was resumed until 4 h post-dose. After administration, approximately 400 μL of blood sample was collected from the orbital sinus at 10 min, 25 min, 40 min, 1 h, 2 h, 3 h, 4 h, 6 h, and 8 h, and placed into heparinized tubes.

### 3.12. Bioanalytical Method

A total of 200 μL of plasma was added to 600 μL of naringenin solution (20 ng/mL) as internal standard. After vortexing for 30 min, the mixtures were centrifuged for 5 min (25 °C, 14,000 rpm). Subsequently, the supernatant was analyzed using LC-MS with a SCIEX Triple QuadTM 4500 instrument from SCIEX, LLC (Framingham, MA, USA). The instrument was equipped with an electrospray ionization (ESI) source operating in negative ion mode. Chromatographic separation was conducted on an Agilent Eclipse Plus C18 column (4.6 × 150 mm, 5 µm). The mobile phase consists of 0.5% acetic acid solution and methanol (44:56, *v*/*v*) at a flow rate of 1.2 mL·min^−1^. The detection was set at 288 nm. Multiple reaction monitoring modes (MRM) were used to perform the detection and quantification, with *m*/*z* 481.00 → *m*/*z* 300.90 for Silybin and *m*/*z* 271.00 → *m*/*z* 151.00 for naringenin. The DAS 2.0 program was used to perform the PK analysis.

## 4. Conclusions

The present study attempts to address the poor solubility and low bioavailability of Silybin. We have successfully obtained a novel cocrystal of Silybin with L-proline. Though the single crystal structure was not obtained, the stoichiometric ratio was confirmed by ^1^H-NMR and HPLC. The cocrystal exhibited significantly enhanced dissolution performance across various pH conditions, particularly at acidic pH levels, where the apparent solubility of the cocrystal was markedly higher than that of raw Silybin. This improvement was further amplified by the inclusion of precipitation inhibitors, such as PVP K30, which helped maintain a supersaturated state for an extended period. The pharmacokinetic studies in rats provided clear evidence of the cocrystal’s superior performance. At the same dosage, the Silybin cocrystal achieved a 16.4-fold increase in C_max_ and a 16.2-fold improvement in AUC_0–8h_ compared to raw Silybin, and also significantly surpassed the bioavailability of the commercial Silybin–phosphatidylcholine complex. In conclusion, this cocrystal offers a promising strategy to improve the therapeutic potential of Silybin, providing a feasible solution to its clinical limitations.

## Figures and Tables

**Table 1:** Pharmacokinetic parameters of Silybin, phosphatidylcholine complex of Silybin, and Silybin cocrystal.

	T_max_ (h)	C_max_ (ng·mL^−1^)	AUC_0–8h_ (ng·h·mL^−1^)
Silybin	0.17	137.94 ± 49.29	88.55 ± 16.46
Phosphatidylcholine complex of Silybin	0.17	1120.17 ± 588.94	569.21 ± 236.93
Silybin cocrystal	0.17	2257.33 ± 914.51	1431.94 ± 882.80

## Data Availability

The original contributions presented in this study are included in the article/Supplementary Materials; further inquiries can be directed to the corresponding author.

## Supplementary Materials

The following supporting information can be downloaded at: https://www.mdpi.com/article/10.3390/ph18010090/s1, Figure S1: (a) ^1^H-NMR spectra overlay of Silybin, L-proline, and their cocrystal, (b) ^1^H-NMR spectra of cocrystal with peak areas labeled at 3.9 ppm and 2.0 ppm; Figure S2: HPLC pattern of the cocrystal; Figure S3: TGA profile of the cocrystal; Figure S4: DVS profile of the cocrystal before and after DVS experiment; Figure S5: XRPD pattern of the sample after 10 min dissolution in three pH buffers; Figure S6: XRPD pattern of the sample after 10 min dissolution in three pH buffers. Figure S7. Dissolution profiles of Silybin cocrystal in the absence and presence of different polymers in pH 2.0 solution. Table S1. Specification of the chemical samples. Table S2. Solubility after equilibrium for 24 h.
