# Supplementary material for: Silybin Cocrystals with Improved Solubility and Bioavailability
Source: Pharmaceuticals (Basel). 2025 Jan 13;18(1):90. doi: 10.3390/ph18010090 (PMC11768837; doi:10.3390/ph18010090)
Supplement: Supplementary file 1 [file pharmaceuticals-18-00090-s001.zip › pharmaceuticals-3349435-supplementary.pdf]

Silybin Cocrystals with Improved Solubility and Bioavailability

Bingqing Zhu, Zhenfeng Ding, Xiaoyi Rong, Shengqiang Li and Xuefeng Mei*

Pharmaceutical Analytical & Solid-State Chemistry Research Center, Shanghai Institute of Materia Medica, Chinese Academy of Sciences, 555 Zuchongzhi Road, Shanghai 201203, China; zhubingq@simm.ac.cn (B.Z.); rongxiaoyi@simm.ac.cn (X.R.)

* Correspondence: xuefengmei@simm.ac.cn

Table S1. Specification of the chemical samples.

Chemical name	Chemical structure	Source	CAS No.	Purity
L-proline	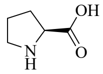	J&K Scientific Ltd. (Beijing, China)	7447-40-7	98.0%
Silybin	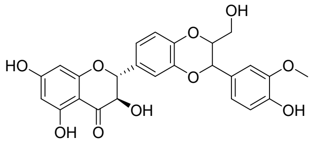	Liaoning Fengrui Natural Biotechnology Co., Ltd. (China)	22888-70-6	95.0%
Silybin complex	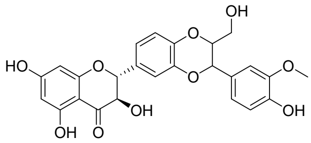 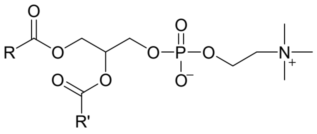 R,R' = fatty acid residues	Indena Pharmaceuticals	Silybin: 22888-70-6 Phospholipids (Lecithins): 8002-43-5	30.0% Silybin
Ethanol	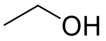	Sinopharm Chemical Reagent Co., Ltd. (Shanghai, China)	64-17-5	-
Water	H_2O	Sinopharm Chemical Reagent Co., Ltd. (Shanghai, China)	7732-18-5	-

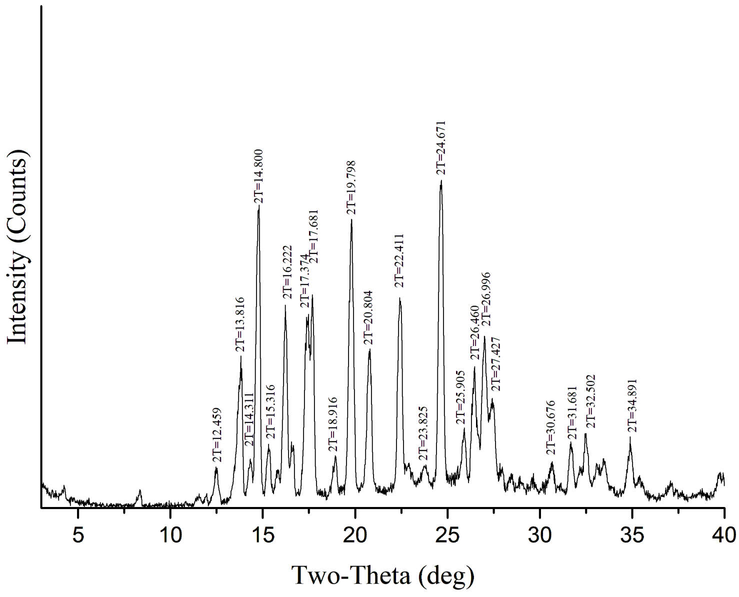
Intensity (Counts)
Two-Theta (deg)
5
10
15
20
25
30
35
40
2T=12.459
2T=13.816
2T=14.311
2T=14.800
2T=15.316
2T=16.222
2T=17.374
2T=17.681
2T=18.916
2T=19.798
2T=20.804
2T=22.411
2T=23.825
2T=24.671
2T=25.905
2T=26.460
2T=26.996
2T=27.427
2T=30.676
2T=31.681
2T=32.502
2T=34.891

(a)

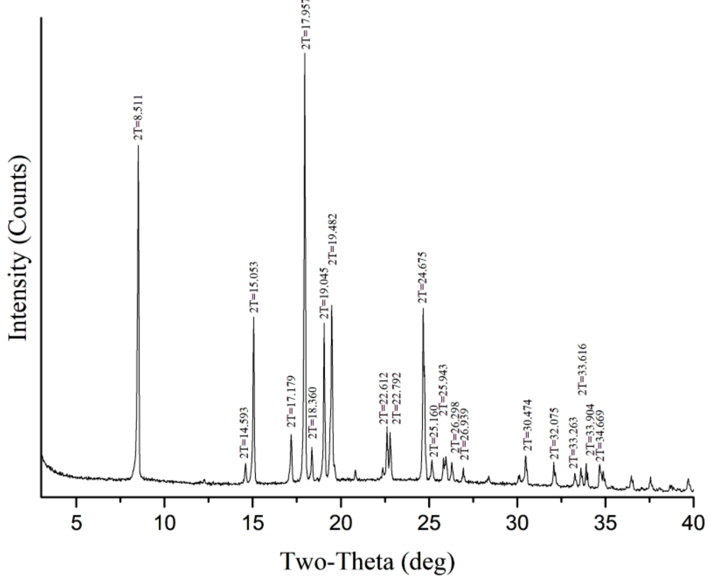
Intensity (Counts)
Two-Theta (deg)
5
10
15
20
25
30
35
40
2T=8.511
2T=14.593
2T=15.053
2T=17.179
2T=17.957
2T=18.360
2T=19.045
2T=19.482
2T=22.612
2T=22.792
2T=24.675
2T=25.160
2T=25.943
2T=26.298
2T=26.939
2T=30.474
2T=32.075
2T=33.263
2T=33.616
2T=33.904
2T=34.669

(b)

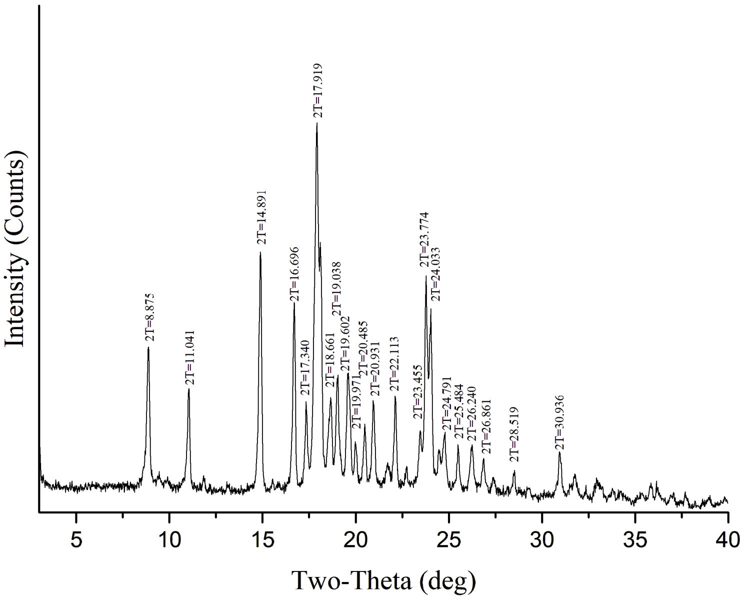

(c)

Figure S1. Peak values in PXRD patterns of (a) Silybin, (b) L-proline, and (c) Cocrystal

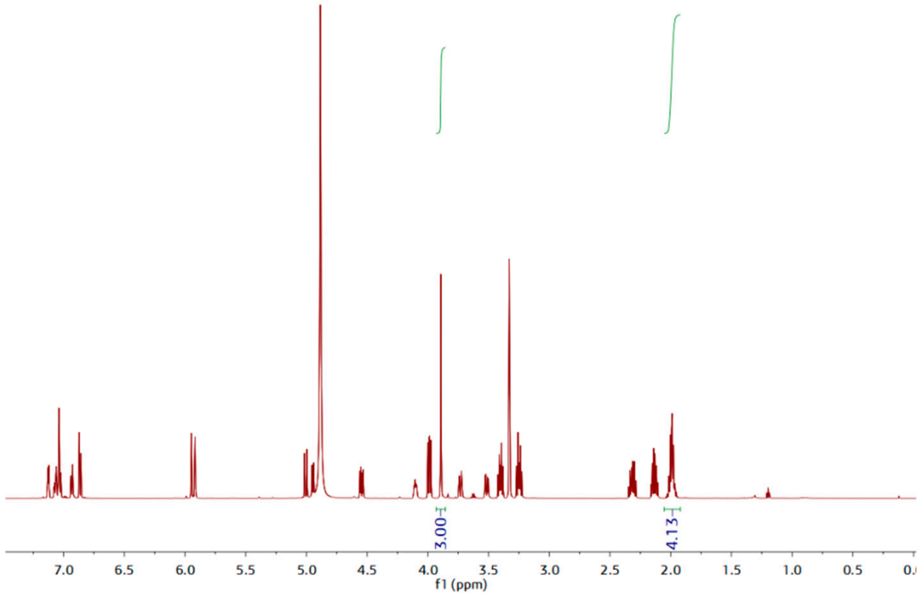

(a)

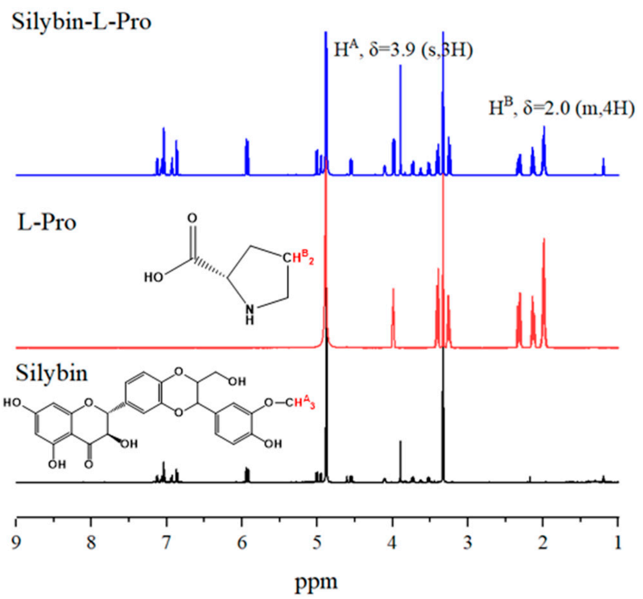

(b)

Figure S2. (a) ^{1}H-NMR spectra of cocrystal with peak areas labeled at 3.89 ppm and 1.99 ppm. (b) ^{1}H-NMR spectra overlay of Silybin, L-proline and the cocrystal.

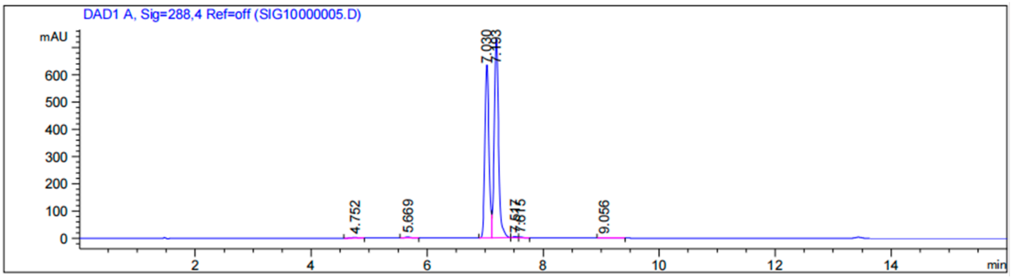

Figure S3. HPLC pattern of the cocrystal.

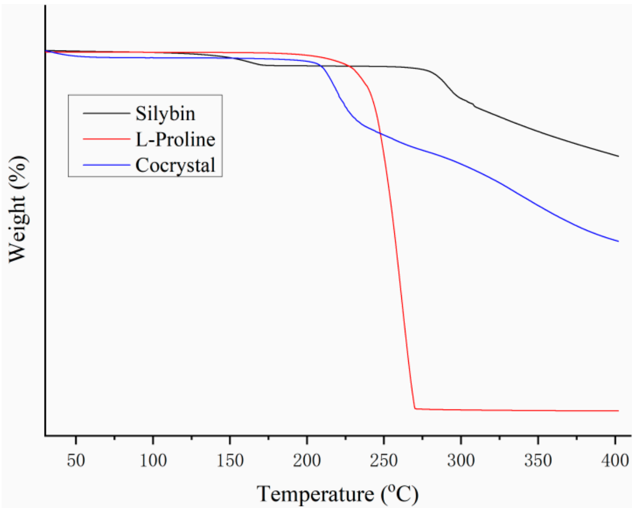

Figure S4. TGA profiles of Silybin, L-proline and cocrystal.

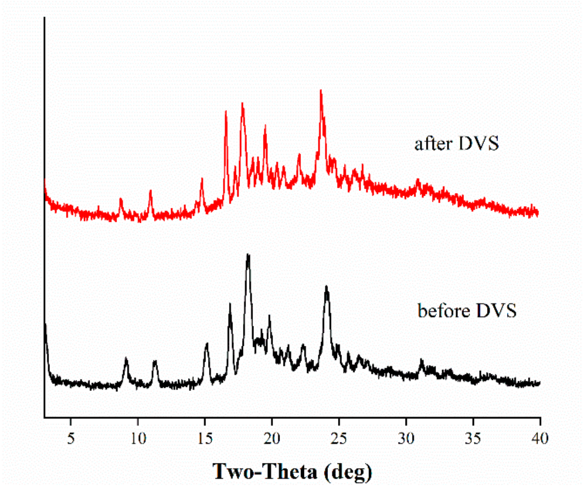

Figure S5. PXRD profile of the cocrystal before and after DVS experiment.

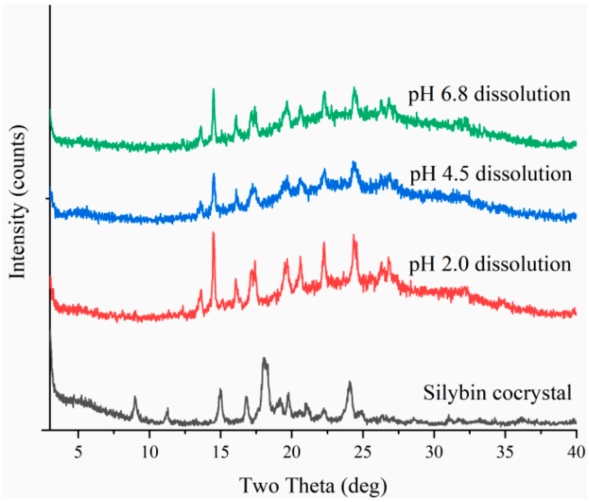

Figure S6. XRPD pattern of the sample after 10 min dissolution in three pH buffers.

Table S2. Solubility after equilibrium for 24 hours.

	Silybin extract (μg/ml)	Silybin cocrystal (μg/ml)	Silybin complex (μg/ml)
pH 2.0	5.8	6.3	18.20
pH 4.5	10.8	10.5	32.1
pH 6.8	42.3	48.5	35.7

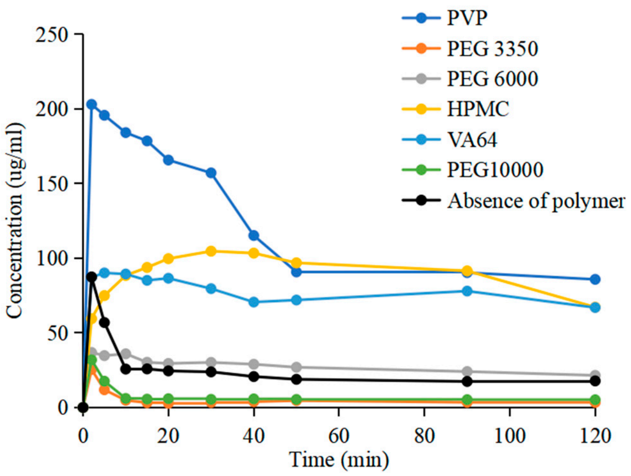

Figure S7. Dissolution profiles of Silybin cocrystal in the absence and presence of different polymers in pH 2.0 solution.
